# Erythrocytes do not activate purified and platelet soluble guanylate cyclases even in conditions favourable for NO synthesis

**DOI:** 10.1186/s12964-016-0139-9

**Published:** 2016-08-11

**Authors:** Stepan Gambaryan, Hariharan Subramanian, Linda Kehrer, Igor Mindukshev, Julia Sudnitsyna, Cora Reiss, Natalia Rukoyatkina, Andreas Friebe, Iraida Sharina, Emil Martin, Ulrich Walter

**Affiliations:** 1 Institute of Clinical Biochemistry and Pathobiochemistry, University of Wuerzburg, Grombuehlstraße 12, D-97080 Wuerzburg, Germany; 2 Sechenov Institute of Evolutionary Physiology and Biochemistry, Russian Academy of Sciences, Thorez pr. 44, St, Petersburg, 194223 Russia; 3 Institute of Experimental Cardiovascular Research, University Medical Center Hamburg-Eppendorf, Hamburg, Germany; 4 Institute of Physiology, University of Wuerzburg, Wuerzburg, Germany; 5 Center for Thrombosis and Hemostasis (CTH), University Medical Center Mainz, Mainz, Germany; 6 Department of Internal Medicine, Division of Cardiology, University of Texas Houston Medical School, Houston, USA; 7 German Centre for Cardiovascular Research (DZHK) RheinMain, Mainz, Germany

**Keywords:** Erythrocytes, Nitric oxide, Soluble guanylate cyclase, Platelets, Hemoglobin

## Abstract

**Background:**

Direct interaction between Red blood cells (RBCs) and platelets is known for a long time. The bleeding time is prolonged in anemic patients independent of their platelet count and could be corrected by transfusion of RBCs, which indicates that RBCs play an important role in hemostasis and platelet activation. However, in the last few years, opposing mechanisms of platelet inhibition by RBCs derived nitric oxide (NO) were proposed. The aim of our study was to identify whether RBCs could produce NO and activate soluble guanylate cyclase (sGC) in platelets.

**Methods:**

To test whether RBCs could activate sGC under different conditions (whole blood, under hypoxia, or even loaded with NO), we used our well-established and highly sensitive models of NO-dependent sGC activation in platelets and activation of purified sGC. The activation of sGC was monitored by detecting the phosphorylation of Vasodilator Stimulated Phosphoprotein (VASP^S239^) by flow cytometry and Western blot. ANOVA followed by Bonferroni’s test and Student’s *t*-test were used as appropriate.

**Results:**

We show that in the whole blood, RBCs prevent NO-mediated inhibition of ADP and TRAP6-induced platelet activation. Likewise, coincubation of RBCs with platelets results in strong inhibition of NO-induced sGC activation. Under hypoxic conditions, incubation of RBCs with NO donor leads to Hb-NO formation which inhibits sGC activation in platelets. Similarly, RBCs inhibit activation of purified sGC, even under conditions optimal for RBC-mediated generation of NO from nitrite.

**Conclusions:**

All our experiments demonstrate that RBCs act as strong NO scavengers and prevent NO-mediated inhibition of activated platelets. In all tested conditions, RBCs were not able to activate platelet or purified sGC.

## Plain English summary

The main role of RBCs in the human body is to transport O_2_ from lungs to different tissues and CO_2_ back to lungs. Recently studies propose that RBCs, under certain conditions, can produce NO and inhibit platelet activation. Nitric Oxide/soluble Guanylate Cyclase/cyclic guanosine monophosphate/protein kinase G (NO/sGC/cGMP/PKG) pathway is one of the most powerful mechanisms responsible for platelet inhibition. However, the activation of platelet sGC/cGMP/PKG by NO released from RBCs was not directly proven.

Therefore, the main aim of our work was to test whether RBCs can release NO that can stimulate platelet sGC. Incubating RBCs with purified sGC and platelets under normal and hypoxic conditions, did not induce sGC activation indicating that NO is not released from RBCs under tested conditions. Rather, externally added NO onto whole blood was scavenged by RBCs, thereby preventing sGC activation in platelets. Our data strongly indicate that RBCs do not release biologically active NO that is sufficient to activate platelet sGC, which is a highly sensitive NO receptor.

## Background

Red blood cells (RBCs) and platelets represent the major cell population in mammalian blood. Functional interactions between RBCs and platelets are known for a long time from clinical observations. The bleeding time is prolonged in patients with anemia independent on their platelet count and could be corrected by RBCs transfusion, and bleeding defects are directly connected with platelet function but not with the blood coagulation system [1–3]. On the other hand, RBCs transfusion could increase platelet activation and might be dangerous in treatment of coronary artery diseases [4]. These clinical observations clearly pointed out that RBCs, by still unidentified mechanism(s), play some important role in platelet activation. However, recently in several papers [5–9] platelet inhibition by RBCs-derived NO was described. In all these papers, platelet inhibition by deoxygenated RBCs, that can reduce nitrite to NO was shown. Importantly, in none of these papers direct activation of platelet soluble Guanylate Cyclase (sGC) by RBCs-derived NO was shown. In papers where the inhibition of platelet function by nitrite/RBCs was described [5–9], platelet activation was assessed by aggregometry and flow cytometry, which is not sufficient to confirm whether the inhibition was due to the activation of NO/cGMP pathway in platelets. Several publications described direct NO production from RBCs by endothelial NO synthase (eNOS) [10, 11], but other studies did not find any functionally active NOS in RBCs [12]. The direct activation of platelet sGC by RBCs was never shown before. Hence, the connection between RBCs, eNOS and hemoglobin (Hb) to platelet sGC activation and platelet inhibition cannot be proven.

Hb and RBCs are involved in the modulation of NO signaling and metabolism. Since the discovery of NO as a signaling molecule, numerous studies proposed the formation of the nitrosyl-heme adduct of Hb (NO-Hb), a source of bioavailable NO. The nitrosylation of conserved cysteine of Hb β-chain (β93Cysteine) by NO and the formation of S-Nitrosohemoglobin (SNO-Hb) were proposed as putative mechanisms for Hb functioning as NO carrier [13, 14]. These interactions between NO and Hb are superseded by the fast reaction between NO and oxyhemoglobin (oxyHb). According to mathematical modelling and experimental data, most of NO in the blood is annihilated by the fast irreversible reaction of oxyHb with NO, which yields oxidized Hb (methemoglobin, metHb) and nitrate [15]. On the other hand, deoxyhemoglobin (deoxy-Hb) may act as a nitrite reductase, promoting the synthesis of NO from inorganic nitrite [16, 17].

The high affinity NO receptor, sGC mediates most of the biological functions of NO, especially at low NO concentrations [18, 19]. Although different reactions between NO and Hb were established, the mechanisms of NO/SNO release from Hb are not well understood. It is less clear how NO can be released from RBCs under physiologic conditions of overwhelming excess of free Hb. It remains to be proved that RBCs-derived NO can activate platelet sGC.

Endogenous NO is a well-known and potent inhibitor of platelet activation (reviewed in [20, 21]). NO-dependent inhibition of platelets is solely mediated by sGC [22]. Intact platelet sGC is highly sensitive to endogenous NO and even nanomolar NO concentrations are sufficient for cGMP generation [23]. In platelets, Protein Kinase G (PKG), which phosphorylates multiple substrates responsible for platelet inhibition, is the major downstream target of cGMP [21]. For evaluation of platelet sGC activity, we developed a highly sensitive method, based on NO/sGC/cGMP/PKG dependent phosphorylation of vasodilator-stimulated phosphoprotein (VASP) in human platelets, which has been used in numerous studies [20]. Most studies that report NO release from RBCs had been performed using blood vessel relaxation experiments or with isolated washed RBCs. However, both approaches were not sufficient to answer the question whether RBCs can produce biologically active NO/SNO. Vasorelaxation is a complex process controlled not only by NO, but also by a number of other vasodilators. Moreover, the experiments with isolated RBCs never demonstrated that NO or other reactive nitrogen species generated by RBCs result in activation of sGC.

In this report, we directly addressed this question by determining the sGC-dependent signaling in intact platelets and the activation of purified sGC. Our data clearly showed that RBCs, under the tested conditions, cannot activate platelet sGC or purified sGC, but in contrast, they act as a potent NO scavengers.

## Methods

### Materials

DEA-NO (Biomol, Hamburg, Germany), iloprost (Ilomedin; Schering, Berlin, Germany), Thrombin Receptor Activating Peptide-6 (TRAP-6) (Bachem, Weil am Rhein, Germany) were purchased. Antibody against actin, L-arginine, L-N^G^-Nitroarginine methyl ester (L-NAME), NG-Methyl-L-arginine (L-NMA), and phenylephrine were obtained from Sigma (Munich, Germany). The PAC-1-FITC and CD-41-FITC antibodies were obtained from BD-Bioscience (Heidelberg, Germany). Antibodies against VASP (Ser^239^) were from Nano Tools (Teningen, Germany). sGC and PKG I antibodies were described previously [24, 25].

### Preparation of human platelets, white blood cells (WBCs) and RBCs

Human platelets were prepared as reported previously [26]. Blood was collected from healthy volunteers, and WBCs were isolated by Leucosep tubes (Greiner, Frickenhausen, Germany) according to the manufacturer’s instructions. WBC concentration was adjusted to 6×10^6^/ml. RBCs were collected after separation of PRP and washed thrice with HEPES buffer; the supernatant from the last wash was used as control to confirm the absence of free Hb. For experiments with RBCs, suspensions which did not contain free hemoglobin were used.

### Platelet aggregation

Aggregation in whole blood was measured by Multiplate (Roche Diagnostics, Mannheim, Germany), in PRP and washed platelets by Apact4004 (LabiTec, Ahrensburg, Germany) aggregometers. Citrated whole blood was preincubated with sodium nitroprusside (SNP) and aggregation was initiated by ADP (10 μM). PRP and washed platelets were preincubated for 1 min with SNP or iloprost and aggregation was initiated by ADP.

### FACS analysis

FACS analysis was performed using a Becton Dickinson FACS Calibur with CELL Quest software, version 3.1f (Becton Dickinson, Heidelberg, Germany). Platelets in the whole blood were identified by CD41 staining. Washed platelets concentration was adjusted to 3×10^8^/ml and for washed platelets/RBC mixture 4.5×10^9^/ml washed RBCs were added to platelet suspension. All samples after stimulation were directly fixed with 3 % formaldehyde. The samples were incubated with PAC-1-FITC, or CD41-FITC antibodies and analysed by FACS. For phospho-VASP, the samples were permeabilized with 0.2 % Triton X-100.

### Western blot analysis

Samples were lysed with Laemmli buffer and the proteins were separated by SDS-PAGE and transferred to nitrocellulose membrane as described [27]. Membranes were incubated with primary antibodies overnight at 4 °C. Goat anti-rabbit or anti-mouse IgG conjugated with horseradish peroxidase were used as secondary antibodies followed by ECL detection. Blots were analysed densitometrically using NIH Image J software for uncalibrated optical density.

### Experiments with deoxygenated washed platelets and RBCs

HEPES buffer was degassed under vacuum and bubbled with argon. This buffer was used for washing and resuspension of washed platelets and RBCs. RBCs and platelets were further bubbled with argon and kept under argon in closed gas-tight tubes. Then, RBCs were washed 3 times with argon-saturated HEPES to wash out free hemoglobin from lysed RBCs. In some experiments deoxy-RBCs were incubated with DEA-NO and/or with NaNO_2_ prior addition to platelet suspension. For experiments with purified sGC, preparations of deoxy-RBC were also transferred into an anaerobic glove chamber (Model 110 V, Coy Laboratory Products, Inc.) and equilibrated for additional 2 h in anaerobic environment.

### Spectral analysis of Hb

To confirm the Hb status of RBCs, UV spectra were taken for oxy-Hb, and deoxy-Hb after incubation with NO donor. 10 μl of RBC were lysed in hypoosmotic media (HEPES buffer diluted by water to 50 mOsm), and the visible absorption spectra in the 450–700 nm range were immediately collected on a UV–vis Spectrophotometer (SPECS SSP-715, Spectroscopy Systems, Russia).

### sGC activity assay

Full-length human sGC was expressed in insect Sf9 cells as described previously [28]. Recombinant sGC was purified using anion exchange and Ni-affinity chromatography as detailed previously [29]. Anaerobic sGC was prepared in an anaerobic train as described previously, [19] and transferred into the anaerobic glove chamber. Enzymatic sGC activity was assayed using a modified [α-^32^P]GTP to [^32^P]cGMP conversion assay [30] with buffers prepared anaerobically and equilibrated in the anaerobic chamber overnight. To measure NO-induced activity, 0.01 μg sGC in 10 mM HEPES (pH 7.4), 150 mM NaCl, 1 mM DTT, 3 mM MgCl_2_, was incubated with indicated RBCs or buffer prior to exposure to the mixture containing NO donor (DEA-NO) and GTP. To determine if RBCs generate any bioavailable NO from nitrite, anaerobic RBCs were incubated at 37^°^C for 15 s or 5 min with indicated amount of nitrite, prior to the addition to 0.1 μg sGC. The cGMP-forming reaction was initiated by 1 mM GTP/[α-^32^P]GTP (~100,000 cpm). The reaction was stopped by zinc carbonate precipitation and processed following the protocol established previously [30].

### Data analysis

All experiments were performed at least thrice, and data are expressed as means ± SEM. Differences between groups were analyzed by ANOVA followed by Bonferroni’s test and Student’s *t*-test as appropriate. *p* < 0.05 was considered statistically significant.

## Results

### RBCs prevent NO donor-mediated platelet response

**Fig. 1:**
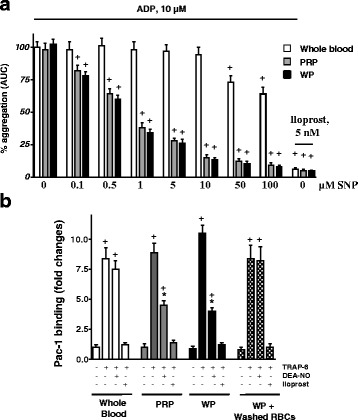
NO-dependent platelet inhibition is blunted in the presence of RBCs. **a** ADP-induced platelet aggregation measured in the whole blood, platelet rich plasma (PRP) and washed platelets (WP). Aggregation in whole blood was measured by Multiplate, in PRP and WP by Apact 4004 aggregometer. For whole blood, 300 μl of citrated whole blood and 300 μl saline were added to the test cell together with indicated concentrations of SNP. After three minutes of incubation at 37 °C, aggregation was initiated by ADP (10 μM). Aggregation curves were recorded for 6 min. PRP and WP samples were preincubated for 1 min with indicated concentrations of SNP or iloprost and aggregation was initiated by 10 μm of ADP and monitored for 3 min. Data of platelet aggregation are presented as area under the curve (AUC). **b** FACS analysis of integrin αIIbβ3 activation (Pac-1 binding) measured in the whole blood, PRP, washed platelets (WP), and WP with washed RBCs (10^9^/ml). Samples were prepared as described under “Materials and Methods”. Data are presented as mean ± SEM, *n* = 4; ^+^ *p* < 0.05, compared to control, *p < 0.05 compared to Whole blood sample preincubated with DEA-NO

**Fig. 2:**
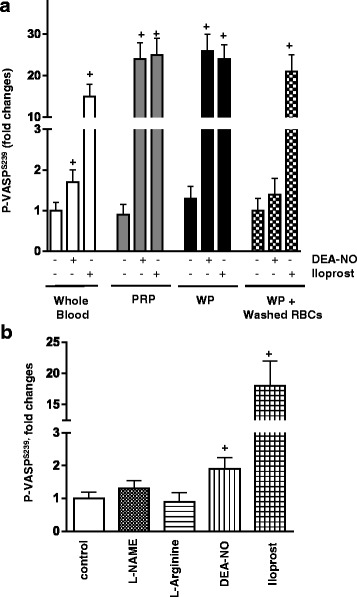
Inhibition of platelet PKG activation in the presence of RBCs. **a** FACS analysis of VASP phosphorylation at S^239^ measured in the whole blood, PRP, washed platelets, and WP with washed RBCs (10^9^/ml). **b** VASP phosphorylation at S^239^ measured in the whole blood in the presence of L-arginine or NOS inhibitor L-NAME (both 200 μM, 10 min preincubation). NO donor (DEA-NO, 5 μM, 2 min) was used as positive control. Samples were prepared as described under “Materials and Methods”. Data are presented as mean ± SEM, *n* = 4; ^+^ *p* < 0.05, compared to control

Inhibition of platelet aggregation by RBCs-generated NO was reported in several publications [5, 7, 11, 31]. To test whether RBCs influence platelet aggregation, we measured ADP-triggered aggregation in whole blood, PRP, and washed platelets. In whole blood, very high (50 and 100 μM) concentrations of Sodium nitroprusside (SNP) were required for partial inhibition of platelet aggregation (25 ± 4 and 32 ± 6 %, respectively). On the contrary, in PRP and washed platelets, 0.5 μM SNP was sufficient to achieve similar degrees of inhibition. Activation of cAMP/Protein Kinase A (PKA) signaling by iloprost (5 nM) completely inhibited platelet aggregation in all the three preparations (Fig. 1a). Corroborating the aggregation studies, NO donor (DEA-NO) significantly inhibited TRAP-6 induced integrin (αIIbβ3) activation in PRP and washed platelets, but not in whole blood or in washed platelets coincubated with washed RBCs. Iloprost, used as a positive control in these experiments, completely prevented integrin αIIbβ3 activation (Fig. 1b). To prove that the platelet inhibitory response was mediated by PKG and PKA activation, we measured VASP phosphorylation on the same sets of experiments. DEA-NO only slightly (1.7 ± 0.3 fold) increased VASP phosphorylation in the whole blood samples, whereas in PRP and washed platelets the increase of VASP phosphorylation was more than 25-fold and comparable with the iloprost-induced response. Coincubation of washed platelets with washed RBCs completely prevented DEA-NO-induced VASP phosphorylation (Fig. 2a). It was recently [31] reported that in RBCs, activation of eNOS by L-arginine has an inhibitory effect on platelet aggregation. However, this study did not determine whether the inhibition is due to the activation of sGC/cGMP/PKG pathway in platelets. To address this issue, we incubated the whole blood with L-arginine or NOS inhibitor (L-NAME) and measured platelet VASP phosphorylation by FACS analysis. Neither inhibition, nor stimulation of NOS in whole blood affected VASP phosphorylation (Fig. 2b).

### Presence of RBCs prevents NO-induced PKG activation in platelets

**Fig. 3:**
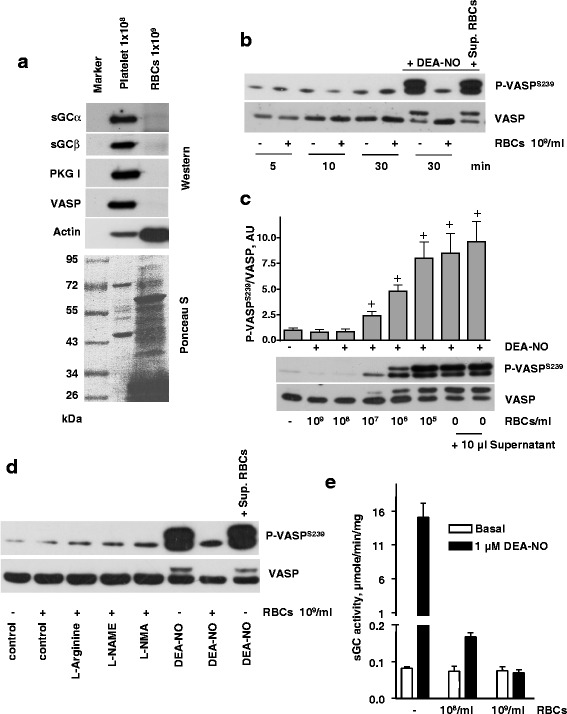
Inhibition of sGC activity by RBCs. **a** Western blot analysis of sGC, PKG, VASP, and actin expression in platelet and RBC lysates. Upper panel represent Western blot and lower panel represent Ponceau-S-stained membrane. **b-d**) Western blot analysis of VASP^S239^ phosphorylation in platelets (3x10^8^/ml) treated with 1 μM DEA-NO in the presence of RBCs (5x10^8^/ml, in 10 μl). **b** Platelets were coincubated with RBCs (10^9^/ml, 10 μl), or the same volume (10 μl) of supernatant from the last wash of RBCs for indicated time. When indicated, samples were treated with DEA-NO (1 μM, 30 min). **c** Indicated amount of RBCs (10 μl) were added to platelet suspension for 10 min, and then stimulated with DEA-NO (1 μM, 1 min). When indicated, the same volume (10 μl) of supernatant from the last wash of RBCs was added to the platelets. For the bar graphs, immunoblots were scanned and quantified by the Image J program. The intensity of the VASP^Ser239^ signal was normalized to the total VASP signal, which was designated as 1 in control samples. Results are means ± SEM, *n* = 4, + significant difference from control samples. **d** Platelets (3x10^8^/ml) were coincubated with RBC (5x10^8^/ml, 10 μl) and treated with L-Arginine, L-NAME, or L-NMA for 10 min. When indicated, samples were treated with DEA-NO (10 μM, 1 min). Total VASP blots served as loading control. Shown are representative blots of three independent experiments. **e** Basal and NO-induced (1 μM DEA-NO) purified sGC activity measured in anaerobic conditions after incubation with buffer or indicated amounts of RBC. Data are presented as mean ± SEM, *n* = 4

In the next series of experiments, we examined whether longer incubation of RBCs with platelets activates platelet PKG. We also assessed the RBC concentration necessary to inhibit NO-dependent activation of PKG. Since VASP phosphorylation by PKG is the primary parameter evaluated in this set of studies, we first tested by Western blotting whether the key elements of the NO/cGMP pathway downstream of eNOS are detectable in RBCs. The lysate from 10^8^/ml platelets exhibited a strong signal for sGC, PKG and VASP. In contrast, these proteins were not detected in RBCs (10^9^/ml) (Fig. 3a).

Next, we observed that incubation of RBCs with platelets in suspension for up to 30 min does not increase the phosphorylation of VASP. Corroborating aggregation experiments, the strong phosphorylation of VASP induced by DEA-NO was completely abolished by RBC (Fig. 3b). The titration of RBCs demonstrated that even 10^8^ RBCs/ml, which is ~50-fold lower than the physiological RBC count, were sufficient to prevent NO-induced phosphorylation of VASP in platelets (Fig. 3c). Neither inhibition, nor stimulation of NOS in RBCs affected PKG activation in platelets (Fig. 3d), which fits in line with the results from whole blood (Fig. 2b).

sGC is a very sensitive receptor for NO, which evolved to respond to nanomolar concentrations of NO [32]. While oxyhemoglobin is a very efficient NO scavenger, the affinity of oxygen-free hemoglobin for NO is comparable to sGC. It was previously shown that deoxygenated RBCs can produce S-nitroso-L-cystein (L-SCNO) and S-nitrosoglutathione (GSNO) [33]. Therefore, we tested whether deoxygenated RBCs affect NO-dependent activation of purified sGC and platelet sGC/PKG pathway. Exogenously added L-CSNO and GSNO are strong activators of purified and platelet sGC (data not shown). However, presence of deoxygenated RBCs had no effect on purified sGC activity and only inhibited DEA-NO-induced sGC activation (Fig. 3e). Similar results were observed, if RBCs were incubated with DEA-NO prior to the addition to sGC (data not shown).

**Fig. 4:**
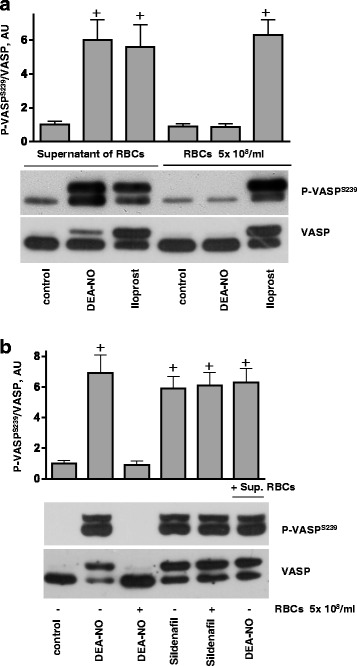
RBCs inhibits platelet PKG stimulated by NO donors, but not by PDE5 inhibition. **a**, **b** Western blot analysis of VASP^S239^ phosphorylation in platelets (3x10^8^/ml) stimulated by DEA-NO (1 μM, 1 min), iloprost (2 nm, 1 min) or sildenafil (1 μM, 5 min) in the presence of washed RBCs (1x 10^8^, 10 μl) or supernatant (10 μl) from last wash of RBCs. **a** Supernatant from RBC (10 μl) washed by HEPES buffer, or RBCs (1x10^8^/ml, 10 μl) were added to platelets for 10 min, then stimulated with DEA-NO, or iloprost. **b** RBCs (10^8^/ml, 10 μl), or supernatant from RBC (10 μl) were added to platelets for 10 min, then stimulated with DEA-NO, or with sildenafil. Total VASP blots served as loading control. Shown are representative blots of 4 independent experiments. For the bar graphs, immunoblots were scanned and quantified by the Image J program. The intensity of the VASP^Ser239^ signal was normalized to the total VASP signal, which was designated as 1 in control samples. Results are means ± SEM, *n* = 4, + significant difference from control samples

Because of the high NO scavenging properties of free Hb [34], special care was taken to assure that the RBC suspensions did not contain free Hb as a result of haemolysis. In all our experiments, we used supernatant from RBCs as a control for the absence of free Hb. In contrast to the addition of RBCs, supernatant from RBCs added to the platelet suspension had no effect on DEA-NO-induced VASP phosphorylation. Also, RBCs and supernatant of RBCs had no influence on PKA-induced VASP phosphorylation stimulated by iloprost (Fig. 4a). Platelet cGMP levels are affected not only by the elevation of cGMP through sGC activation, but also by the inhibition of cGMP-degrading phosphodiesterases. Phosphodiesterase 5 (PDE5) is the main PDE responsible for cGMP degradation in platelets [21]. Therefore, we tested whether RBCs may cause inhibition of PDE5 in platelets. The PDE5 inhibitor sildenafil induced similar VASP phosphorylation in platelet suspension with and without added RBCs (Fig. 4b) indicating that platelet PDE5 was not affected by RBCs.

### Neither deoxygenation, nor addition of nitrate promote RBC-induced sGC activation

**Fig. 5:**
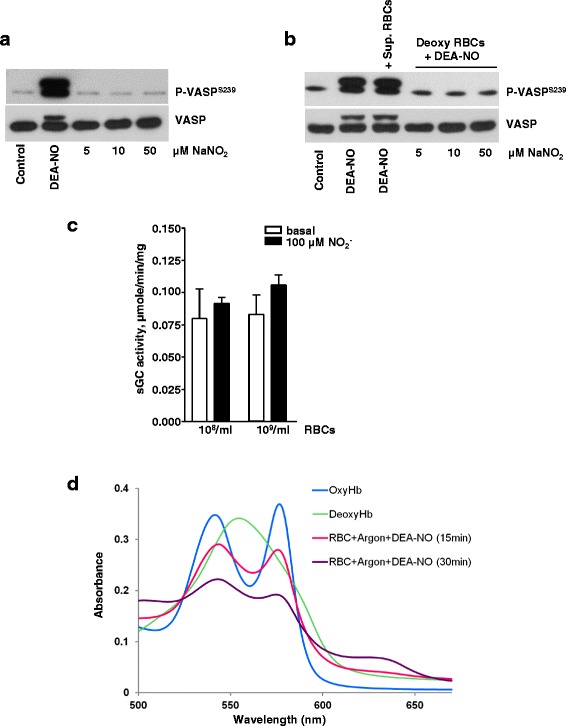
Deoxygenated RBCs loaded with NO did not stimulate platelet PKG. **a** Western blot analysis of VASP^S239^ phosphorylation in platelets (3x10^8^/ml) stimulated by DEA-NO (1 μM, 1 min), or incubated (10 min) with indicated concentrations of NaNO_2_. Experiment was done under argon. **b** Washed platelets and RBCs were deoxygenated by argon. RBCs (1x 10^8^/ml) were washed 3 times by deoxygenated HEPES buffer, deoxygenated for additional 30 min and then incubated with DEA-NO (50 μM, 30 min) and added (1x10^8^/ml in 10 μl) to platelets together with indicated concentrations of NaNO_2_ for 10 min. 10 μl of supernatant from the last wash was used as control for free Hb. Total VASP served as loading control. Shown are representative blots of four independent experiments. **c** sGC activity was measured under anaerobic conditions after incubation with 100 μM sodium nitrite and indicated amount of RBCs, as described in Method section. Data are presented as mean ± SEM, *n* = 4. **d** UV-visible spectra of oxyHb, deoxyHb, and deoxyHb loaded with NO. Hb spectra were taken from, oxygenated, deoxygenated (under argon), or deoxy-RBCs incubated for 15 and 30 min with 50 μM DEA-NO. Shown are representative spectra from four independent experiments

The nitrate-nitrite-NO reductive pathway is an alternative source for NO, which plays a significant role in bacteria and plants [35]. In mammals, the oxygen-independent nitrite reductive pathway was implicated in hypoxic vasodilation [36–38] and control of blood pressure. Some studies showed that RBCs promote nitrite/NO-dependent vasodilation and inhibition of platelet aggregation under hypoxic conditions [7, 39, 40]. We recently showed that incubation of platelets with carbonic anhydrase purified from bovine RBCs in the presence of nitrite increases platelet cGMP content and induces VASP phosphorylation [41]. Therefore, it was important to test whether intact RBCs containing Hb in different status (oxyHb, deoxyHb, NO-Hb), with or without nitrite, and normal or deoxygenated platelets, can activate platelet sGC. Incubation of platelets with different concentrations of nitrite had no effect on PKG-dependent phosphorylation of VASP (Fig. 5a). These results on human platelets are consistent with a report on mouse platelets that nitrite triggers VASP phosphorylation only in the presence of PDE5 inhibitor [42]. Next, we co-incubated deoxygenated RBCs loaded with NO (deoxy RBC + DEA-NO) with deoxygenated platelets in the presence of increasing nitrite concentrations. Even under these conditions, we found no activation of platelet PKG (Fig. 5b). We tested all possible combinations of RBCs containing Hb in different status (oxyHb, deoxyHb, NO-Hb), with or without nitrite, and normal or deoxygenated platelets, and found no evidence of activated platelet PKG (Fig. 5a, b, and other data not shown). To confirm that intact RBCs in the presence of nitrite do not produce NO, we incubated deoxygenated RBCs with nitrite and measured the activity of purified sGC. Anaerobic RBCs preincubated with 100 μM nitrite did not elevate purified sGC activity (Fig. 5c), which is consistent with strong NO-scavenging effect of RBCs. The same results were observed when the experiments were performed under aerobic conditions or with 10 μM nitrite (data not shown). To confirm the Hb status of RBCs, UV spectra were taken from each sample (Fig. 5d). Each spectrum corresponds to well-characterised Hb pattern of oxyHb (RBC), deoxyHb (RBC + Argon), NO-Hb (RBC + Argon + DEA-NO) and visible amount of metHb after 30 min incubation with DEA-NO [43].

### Endothelial cell-derived NO is the main source of platelet sGC activation

**Fig. 6:**
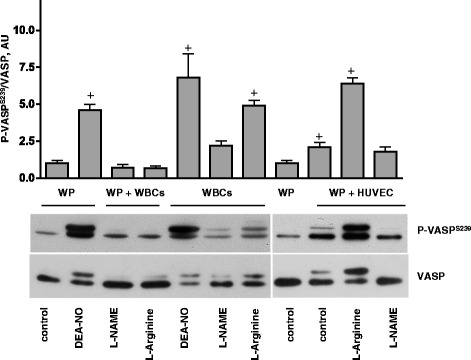
Endothelial cell-derived NO is the only source of platelet PKG activation. Western blot analysis of VASP^S239^ phosphorylation in platelets (3x10^8^/ml). Washed platelets were coincubated with WBCs (7x10^6^/ml) for 10 min, or added to the confluent HUVECs cultured in 24 well plate for 10 min then L-arginine or L-NAME (200 μM each, 10 min) were added to the samples. After 10 min, incubation, WBCs were separated from platelets by centrifugation and suspensions of non-adhered platelets were collected from HUVECs for Western blot analysis. As a control washed platelets and WBCs (7x10^6^/ml) alone were stimulated with DEA-NO (1 μM, 1 min). In addition, WBCs were incubated with the same concentration/time with L-Arginine and L-NAME. Shown are representative blots of four independent experiments. Total VASP served as loading control. For the bar graphs, immunoblots were scanned and quantified by the Image J program. The intensity of the VASP^Ser239^ signal was normalized to the total VASP signal, which was designated as 1 in control samples. Results are means ± SEM, *n* = 4, + significant difference from control samples

In blood vessels, endothelial cells or circulating lymphocytes express NOS isoforms and may potentially be the source of NO for activation of platelet sGC/cGMP/PKG pathway. To evaluate the contribution of endothelial cells and lymphocytes, we co-incubated washed platelets with washed WBCs, or human umbilical vein endothelial cells (HUVECs) in the presence of L-arginine or L-NAME. Incubation of platelets with WBCs in the presence of L-arginine did not activate platelet PKG, however, NOS activity of WBCs was slightly increased by L-arginine and inhibited by L-NAME. It is likely that the amount of NO produced by WBCs in our experimental setting was not sufficient for activation of platelet sGC. In contrast, NO producted by HUVECs, which can be inhibited by L-NAME, strongly stimulates platelet sGC (Fig. 6) suggesting that endothelial cell-derived NO is the main source of platelet sGC activation. Our *in vitro* data are in good agreement with *in vivo* mouse models, where it was shown that eNOS of endothelial cells exerts a direct anti-thrombotic effects on platelets [44].

## Discussion

The key role of RBCs in hypoxic vasodilation is well known. RBCs trigger sGC activation in the smooth muscle cells of the vessel wall that leads to vessel dilation. Few publications report that RBCs release NO during hypoxia, thus activating sGC. Several mechanisms were proposed to argue the NO carrying properties of RBCs/Hb. In one mechanism, Hb nitrosyl-heme adduct (NO-Hb) is regarded as a form of NO storage and carrier. NO released from NO-Hb under hypoxic conditions results in vasodilation [36]. According to another proposed mechanism, S-nitrosylated Cys93 of the Hb β-chain is an equally important source of NO/SNO [45]. However, later studies on mice with mutated β93Cys demonstrated that Cys93-Hb is not important for RBC-induced vasodilation [13, 14]. Finally, NO reduced from nitrite by deoxyhemoglobin was considered as an explanation for hypoxic vasodilation [39, 40, 46]. However, this hypothesis was strongly criticized by others [15]. Strong NO scavenging properties of oxyhemoglobin in hypoxic condition is considered to be an important requirement for effective nitrite/RBCs-dependent NO generation or release. However, the link between RBCs and sGC activation is clearly missing because direct activation of sGC by NO or SNO released from RBCs is not experimentally shown. Nevertheless, until now, NO released from RBCs is considered as one of the established mechanisms for RBC-induced hypoxic vasodilation and platelet inhibition. As RBCs-induced vasodilation might be controlled by other NO-independent mechanisms, we focused our study on the effects of RBC on platelet and purified sGC.

The main aim of our study was to establish whether RBCs could promote sGC activation by releasing NO/SNO under any possible conditions (in whole blood, under hypoxia, or even loaded with NO). To answer the question whether RBCs can activate sGC, we used our well-established model of NO-dependent sGC/cGMP/PKG activation in platelets and NO-mediated activation of purified sGC. Both these systems are highly sensitive to endogenous NO or SNO (GSNO, CysNO) and can be used as a model to answer our question. Previous estimations indicate a high number of molecules of sGC (3700), PKG (3500) and VASP (44000) per single platelet [47]. This high concentration of NO receptor and downstream signaling molecules makes platelets a very sensitive model for detection of NO-dependent sGC activation. PKA and PKG phosphorylate VASP at serines 157 and 239, respectively. In platelets, PKA and PKG can equally phosphorylate both sites. We argue that if RBCs-released NO/SNO is sufficient to induce vasodilation, platelet sGC must be activated in the RBCs/platelet suspension. Only one paper [7] showed more than 3 times increase of platelet cGMP concentration after 5 min incubation with 0.1 μM nitrate, and addition of RBCs further increases cGMP more than 5 times. In this paper, cGMP was measured by the enzyme-linked immunoassay from Cayman Chemicals. It is important to mention that such a high cGMP increase (comparable with NO donors effect) should undoubtedly activate platelet PKG, which was not shown in this manuscript. We specifically addressed this question and used several cGMP determination assays from different suppliers, however we were not able to reproduce their results. Apparently, nitrite itself, and especially RBCs, interfere with cGMP assays and, in some cases, might give erroneous increase of platelet cGMP. After summarizing results of eight experiments from different assays, we did not detect any statistically significant cGMP accrual in platelets incubated with nitrite and RBCs. It should be mentioned that direct measurement of cGMP levels in platelets, is complex and prone to artefacts, therefore we did not include these data. This question has been described in details in our recent review [48]. VASP phosphorylation at Ser239 mediated by PKG, which is even more sensitive than determination of cGMP itself [49] is now accepted as one of the most commonly used standards for evaluation of sGC/cGMP/PKG system activation, especially in platelets. Therefore, in all our experiments we used VASP Ser239 phosphorylation as a readout of PKG activation mediated by cGMP increase. We also tested whether RBCs activate purified sGC, which is sensitive to low NO/SNO concentrations [29]. The experiments presented in the current report clearly demonstrated that RBCs did not directly stimulate purified sGC, under normoxic or hypoxic conditions or in the presence of nitrite. Moreover, RBCs at physiological concentration, completely inhibited sGC activation even by micromolar concentrations of NO donor (Fig. 3b-e). Washed RBCs do not express detectable amount of sGC, PKG, and VASP (Fig. 3a). Therefore, in the experiments with RBCs/platelet suspension, the phospho-VASP signal reflects the activation of sGC in platelets. These experiments also show lack of platelet sGC activation in the presence of RBCs and nitrite.

We should emphasize that thorough washing of RBCs is required because of the strong NO-scavenging by free Hb released from lysed RBCs. In about 1/3 of our experiments (these data were excluded) the supernatant of RBCs, which was always used as a control for free Hb contamination, inhibited NO-mediated VASP phosphorylation. It should be noted that in all papers describing inhibition of platelet activation by RBCs [5–7, 50], the results were assessed by aggregometry. In such experimental setting, RBCs-induced effects other than NO/cGMP-mediated platelet inhibition cannot be ruled out.

The experiments performed with whole blood (Fig. 2b) or with RBC/platelet suspension (Fig. 3d) demonstrated that stimulation/inhibition of NOS had no effect on platelet sGC activation. Our studies demonstrated that L-arginine-stimulated NO synthesis in WBCs is sufficient for sGC activation in WBCs, but not of platelet sGC (Fig. 6). However, these data do not exclude that under other experimental conditions WBC-produced NO could activate platelet sGC as well.

RBCs and platelets are the major cell populations in blood, however until now little is known about interactions between these two cell types. RBCs are involved in thrombus formation, but it is not clear which functional role they play in the development of thrombosis [51]. There are no indications in the literature concerning direct platelet/RBCs interactions. In vivo, circulating platelets are continuously exposed to activating and inhibiting factors. RBCs are one of the sources of platelet activating factors like ATP, ADP and Hb released from injured RBCs, which scavenges NO. Hemolysis of RBCs which leads to increase of free Hb, ADP and ATP in plasma is the main reason of platelet hyperreactivity in sickle cell disease patients [52, 53]. On the other hand, endothelial cells derived NO and prostacyclin are the major platelet inhibitory factors. A well-regulated equilibrium between these two opposing processes is important for the normal hemostasis and an impairment of this equilibrium will promote thrombotic or bleeding disorders. Our experiments support the clinical data [1–3] that RBCs play only stimulatory role in platelets and they are not involved in platelet inhibitory pathways.

## Conclusions

All our data demonstrated that RBCs act as strong NO scavengers and prevent NO-mediated inhibition of platelet. In all tested conditions, RBCs were not able to activate platelet or purified sGC.

## Abbreviations

deoxy-Hb, deoxyhemoglobin; Hb, hemoglobin; HUVECs, human umbilical vein endothelial cells; L-NAME, L-N^G^-nitroarginine methyl ester; L-NMA, NG-Methyl-L-arginine; L-SCNO, S-nitroso-L-cystein; RBCs, red blood cells or erythrocytes; sGC, soluble guanylate cyclase; NO, nitric oxide; VASP, vasodilator Stimulated Phosphoprotein; NO-Hb, nitrosyl-heme adduct; oxyHb, oxyhemoglobin; metHb, methemoglobin; PKG, protein kinase G; PKA, protein kinase A; GSNO, S-nitrosoglutathione; SNP, sodium nitroprusside
